# Cost Comparison of Open and Arthroscopic Treatment Options for SLAP Tears

**DOI:** 10.1016/j.asmr.2020.09.020

**Published:** 2021-01-30

**Authors:** Lambert T. Li, Carlin Chuck, Steven L. Bokshan, Steven F. DeFroda, Brett D. Owens

**Affiliations:** Department of Orthopaedic Surgery, Brown University, Warren Alpert School of Medicine, Providence, RI

## Abstract

**Purpose:**

To identify cost drivers of open biceps tenodesis, arthroscopic biceps tenodesis, and arthroscopic SLAP repair in the setting of isolated SLAP tears and to perform a direct cost comparison between the procedures.

**Methods:**

The 2014 State Ambulatory Surgery and Services Databases from 6 US states were used. Cases with Current Procedural Terminology codes 23430 (tenodesis of long tendon of biceps), 29807 (shoulder arthroscopy, repair of SLAP lesion), and 29828 (shoulder arthroscopy, biceps tenodesis) were selected, excluding patients who were >50 years old or had a concomitant rotator cuff repair. Generalized linear models were used to model costs based on surgical and patient variables.

**Results:**

The mean patient age was 41.8 years for open biceps tenodesis, 31.6 for arthroscopic SLAP repair, and 41.3 for arthroscopic biceps tenodesis (*P* < .001). Open biceps tenodesis had cost reductions of $5,664 over arthroscopic biceps tenodesis (*P* = .001) and $2,320 over arthroscopic SLAP repair (*P* = .043). Male sex was associated with $3,798 more in costs (*P* < .001), presence of ≥1 comorbidities added $1,829 (*P* = .002), and each minute in the operating room added $37 (*P* < .001). Operative time for open biceps tenodesis averaged 114 minutes, and both arthroscopic procedures averaged 94 minutes (*P* < .001). Low-volume facilities were associated with $5,536 higher costs for arthroscopic biceps tenodesis (*P* = .001).

**Conclusion:**

In patients aged ≤50 years with isolated SLAP tears, open biceps tenodesis provides cost savings over arthroscopic methods of treatment. There was no significant cost difference between arthroscopic SLAP repairs and arthroscopic biceps tenodesis. Given the increased emphasis on cost containment, surgeons should be aware of the procedural costs associated with the treatment of SLAP tears.

**Level of Evidence:**

III, retrospective cohort study.

Superior labral injuries are common in the United States and are frequently treated with superior labrum anterior to posterior (SLAP) repair or bicep tenodesis, particularly in the younger, active population.^1^, ^2^, ^3^ Although both SLAP repair and bicep tenodesis have been shown to provide a high degree of efficacy,^4^^,^^5^ it is unclear what the long-term effects are, if any, of shifting the biceps tendon as opposed to performing an anatomic labral repair.^6^, ^7^, ^8^

Recent meta-analyses have showed mixed conclusions regarding the superiority of either procedure. Some meta-analyses have demonstrated higher clinical postoperative scores and return to preinjury sports participation favoring biceps tenodesis,^9^, ^10^, ^11^ whereas other studies have suggested no difference between the 2 procedures.^12^^,^^13^ Although arthroscopic SLAP repair remains the more common procedure, primary biceps tenodesis is becoming an increasingly common procedure, especially in older age groups.^14^^,^^15^

In a time of ever-growing health care costs, areas where cost savings can be achieved must be identified.^16^ As the optimal treatment for SLAP tears is unclear, costs should be compared between the various surgical options. The economic impacts of primary SLAP repair and bicep tenodesis have been indirectly studied previously.^17^, ^18^, ^19^ A Markov cost-effectiveness model by Paoli et al.^20^ has shown that biceps tenodesis may be more cost-effective than SLAP repair, particularly when considering the cost burden of revision surgery if the repair fails. However, it is unclear what the specific cost drivers of these procedures are. The purpose of this study was to identify cost drivers of open biceps tenodesis, arthroscopic biceps tenodesis, and arthroscopic SLAP repair in the setting of isolated SLAP tears and to perform a direct cost comparison between the procedures. We hypothesized that open biceps tenodesis would be the least costly procedure and arthroscopic SLAP repair would be the most expensive.

## Methods

### Data Source

The State Ambulatory Surgery and Services Database (SASD) from 2014 was used as a data source. Databases from 6 states were used: Florida, Iowa, Kentucky, Maryland, Nevada, and New York. These states were selected to give a cross-regional representation and have been validated in prior cost analyses for orthopedic procedures.^21^, ^22^, ^23^ The SASD is one of the databases under the Healthcare Cost and Utilization Project (HCUP) and has been used as a data source to study many procedures.^21^^,^^23^, ^24^, ^25^, ^26^, ^27^ Encounter-level data on outpatient surgeries is collected for the SASD. More than 200 variables about patient demographics, procedural codes, and surgical variables are included for each case.

### Data Collection

Cases that contained Current Procedural Terminology (CPT) codes 23430 (tenodesis of long tendon of biceps), 29807 (shoulder arthroscopy, repair of SLAP lesion), and 29828 (shoulder arthroscopy, biceps tenodesis) were initially selected. Any patients who had >1 of the aforementioned CPT codes (n = 2), had concomitant arthroscopic rotator cuff repair (CPT code 29827, n = 2,802), did not have a superior glenoid labrum lesion diagnosis (n = 16,400), or were older than 50 years (n = 1,919) were excluded. Superior glenoid labrum lesion diagnosis was based on *International Classification of Diseases, 9th Revision* (ICD-9) code 840.7. None of the open biceps tenodesis cases were associated with the CPT code for diagnostic arthroscopy (29805).

The outcome variable used in this study was total charges in 2014 US dollars. Previous HCUP studies have shown total charges to be a useful proxy measure for estimating costs of surgery.^21^, ^22^, ^23^^,^^25^

### Statistical Analysis

For each procedure, demographic and surgical variables were first assessed for significance under bivariate analysis. Demographic variables included patient race, age, sex, primary insurance, and presence of ≥1 medical comorbidity. Comorbidities are automatically calculated in the SASD based on ICD-9 codes and include chronic conditions requiring ongoing intervention such as diabetes, hypertension, chronic heart disease, and malignancies.^28^ Age and operative time were included as continuous variables, and all other factors were categorical variables. Surgical variables that were assessed included operative time, postoperative hospital admission, number of suture anchors used, and surgical facility volume. Suture anchor usage was determined using Healthcare Common Procedure Coding System (HCPCS) code C1713 (anchor or screw, bone/bone, or bone/tissue). Facility volume was divided into high- and low-volume groups, and facilities that had ≥100 cases per year were considered to be high volume. It should be noted that data on anesthesia method and ownership of the surgical facility were available for some cases in our sample (1,069 and 2,674 cases, respectively). We determined that the cases missing data for these variables may provide bias, as the mean cost differed between cases with data and those missing it. Therefore, we did not include these variables in our analysis.

All variables that were significant under bivariate analysis (*P* < .05) were then included in a generalized linear model with total charges as the outcome, controlling for all other significant patient and surgical factors. Procedure cost was directly compared by analyzing all cases together under a generalized linear model with procedure type included as a variable. All *P* values <.05 were considered to be significant (SPSS Statistics version 25.0; IBM, Armonk, NY).

## Results

After exclusions, there were 333 open biceps tenodesis cases, 4,100 arthroscopic SLAP repair cases, and 242 arthroscopic biceps tenodesis cases. Open biceps tenodesis procedures required significantly more time in the operating room (OR), with a mean operative time of 114 minutes compared with 94 minutes for both arthroscopic SLAP repair and arthroscopic biceps tenodesis (*P* = .001). Across all procedures, cases performed at high-volume facilities averaged 90.2 minutes in operative time compared with 99.3 minutes at low-volume facilities (*P* = .001). Patient age ranged from 18 to 50 years in each group. Mean age was 41.8 years for patients undergoing open biceps tenodesis, 31.6 for patients undergoing arthroscopic SLAP repair, and 41.3 for patients undergoing arthroscopic biceps tenodesis (*P* < .001).

### Open Biceps Tenodesis

On bivariate analysis, surgical facility volume and operative time were associated with higher cost and thus were entered into a generalized linear model (Tables A1 and A2). This model showed that operative time was the only significant predictor of cost for open biceps tenodesis cases (Table 1). Each additional minute in the OR added $56 (*P* < .001). When controlling for operative time, surgical facility volume was not a significant predictor of cost.

**Table 1:** Generalized linear model of all significant factors for open biceps tenodesis

Variable	β	Standard Error of the Mean	95% Confidence Interval		*P* Value
			Lower	Upper	
Intercept	$7,122	$2,526	$2,172	$12,072	.005
Time in operating room	$56	$17	$22	$89	.001

Included but not significant: surgical facility volume.

### Arthroscopic SLAP Repair

On bivariate analysis of arthroscopic SLAP repair, there were significant associations between cost and patient sex, race, insurance, presence of comorbidity, operative time, number of anchors used, and postoperative admission to the hospital (Tables A1 and A2). These variables were included in a generalized linear model (Table 2). This model showed that while controlling for other significant factors, female sex was associated with $3,524 lower costs (*P* < .001). Presence of ≥1 comorbidity was associated with an additional $1,411 of cost (*P* = .019). Each additional minute in the OR added $36 (*P* < .001). Each suture anchor added $1,314 (*P* = .002). Variables included in this regression that were not significant included postoperative hospital admission, patient race, and insurance.

**Table 2:** Generalized linear model of all significant factors for arthroscopic SLAP repair

Variable	β	Standard Error of the Mean	95% Confidence Interval		*P* Value
			Lower	Upper	
Intercept	$12,107	$682	$10,770	$13,444	<.001
Female sex	–$3,524	$685	–$4,866	–$2,181	<.001
Comorbidity present	$1,411	$600	$234	$2,587	.019
Time in operating room	$36	$6	$24	$47	<.001
Number of anchors	$1,314	$431	$470	$2,159	.002

Included but not significant: hospital admission, patient race, insurance.

### Arthroscopic Biceps Tenodesis

Bivariate analysis of arthroscopic biceps tenodesis showed that patient sex and surgical facility volume had significant associations with cost (Table A1). When included in a generalized linear model, both of these variables held significance (Table 3). Female sex was associated with $4,887 lower costs (*P* = .005). High-volume surgical facilities were also associated with a cost reduction of $5,536 over low-volume facilities (*P* = .001).

**Table 3:** Generalized linear model of all significant factors for arthroscopic biceps tenodesis

Variable	β	Standard Error of the Mean	95% Confidence Interval		*P* Value
			Lower	Upper	
Intercept	$25,901	$1,324	$23,306	$28,497	<.001
Female sex	–$4,887	$1,757	–$8,331	–$1,444	.005
High facility volume	–$5,536	$1,673	–$8,814	–$2,258	.001

### Cost Comparison Model

On analysis of cases across all 3 procedures, significant variables under bivariate analysis included hospital admission, patient sex, race, insurance, presence of comorbidity, operative time, and procedure type (Tables A2 and A3). Many of these variables held significance in the generalized linear model (Table 4). Female sex was associated with $3,678 lower costs (*P* < .001). Presence of ≥1 comorbidity added $1,441 in costs (*P* = .013). Each additional minute in the OR added $38 (*P* < .001). Each suture anchor added $1,246 (*P* = .004). While controlling for all of these predictors, the arthroscopic procedures were associated with higher costs. Arthroscopic SLAP repair added $2,281 compared with open biceps tenodesis (*P* = .045). Arthroscopic biceps tenodesis was $5,922 more expensive than open biceps tenodesis (*P* < .001). Variables included in the generalized linear model that were not significant included hospital admission, patient race, and insurance.

**Table 4:** Generalized linear model of all significant factors for surgical management of SLAP tears

Variable	β	Standard Error of the Mean	95% Confidence Interval		*P* Value
			Lower	Upper	
Intercept	$9,683	$1,302	$7,130	$12,235	<.001
Female sex	–$3,678	$659	–$4,969	–$2,386	<.001
Comorbidity present	$1,441	$583	$298	$2,584	.013
Time in operating room	$38	$5	$27	$49	<.001
Number of anchors	$1,246	$433	$397	$2,094	.004
Arthroscopic BT	$5,922	$1,627	$2,733	$9,111	<.001
Arthroscopic SLAP repair	$2,281	$1,139	$48	$4,514	.045
Open BT (reference)	0				

Included but not significant: hospital admission, patient race, insurance.

## Discussion

Our results demonstrate that when controlling for surgical and patient variables that affect cost, open biceps tenodesis was associated with lower costs compared with arthroscopic biceps tenodesis or SLAP repair by $5,922 and $2,281, respectively. The cost difference between the 2 arthroscopic procedures was not significant, as the confidence intervals for these procedures overlapped in our multivariate model. Although Paoli et al.^20^ had previously performed cost-effectiveness modeling using a Markov model for SLAP tears, the cost differences of this investigation were predicated on hypothetical revision rates. The present study expands on this previous work by using actual charge data instead of Medicare fee schedules. Furthermore, as this investigation was not limited to the Medicare fee schedule, a broader and younger age range was included, which ultimately allowed for the assessment of specific cost drivers for each procedure. Although both of the arthroscopic procedures were an average of 20 minutes shorter than open biceps tenodesis, our cost analysis found that open biceps tenodesis was the least costly procedure, similar to the cost modeling performed by Paoli et al.^20^ This was true without considering the need for revision surgery, however. Although it is not completely known why open biceps tenodesis was the least costly procedure independent of operative time, the additional arthroscopic equipment requirements may contribute to this cost differential.

For both arthroscopic SLAP repairs and arthroscopic biceps tenodesis, female sex was associated with lower overall costs. This may be due to an association with injury severity, although there was no difference in mean number of anchors used between men and women in our study. It is well known that SLAP tears are more common in men, in both active populations and the general population.^29^, ^30^, ^31^ However, less is known about the relative severity of these tears. Future studies should assess associations between sex and injury severity, as this study shows that male patients have higher costs. Notably, age was not found to be a significant cost driver for any of the procedures, although the mean age was different between the open and arthroscopic procedures. It is known that the mean age of patients undergoing SLAP repairs is decreasing, so surgeons may reserve tenodesis procedures for older patients.^32^ The cost differences found in this study may help inform surgeons in their decision making around procedure selection.

Several clinical variables were also associated with increased costs. Longer operative times increased costs by $56 per minute for open biceps tenodesis and by $36 per minute for arthroscopic SLAP repair, with overlapping confidence intervals indicating that the cost per minute for these procedures was not significantly different. The open biceps tenodesis cases took 20 minutes longer than either arthroscopic procedure. This study shows that although longer operative time is a cost driver for open biceps tenodesis, the open procedure is still associated with lower overall costs. Moreover, operative time is also a cost driver for arthroscopic SLAP repair. Presence of ≥1 medical comorbidity was also associated with higher costs for arthroscopic SLAP repairs. Comorbidities likely add cost as a result of the increased or more complex perioperative care required for these patients. We also found that use of more suture anchors added to the cost for arthroscopic SLAP repairs. This is intuitive, as anchors are specialized orthopedic implants and have been shown to be drivers of cost in arthroscopic rotator cuff repair and anterior cruciate ligament (ACL) reconstruction, as have longer operative times and patient comorbidities.^21^^,^^23^

High-volume surgical facilities had lower costs for arthroscopic biceps tenodesis. This indicates that experience of the surgical team is an important consideration for this procedure. Mean operative time was also 9 minutes shorter at high-volume facilities. It has been previously shown that high-volume surgical facilities have lower costs in arthroscopic rotator cuff repair.^22^ The same trend was found for total hip and total knee arthroplasties performed in high-volume hospitals.^33^ Similarly, Scott et al.^34^ found that high-volume surgeons had lower costs for ACL reconstruction and total shoulder arthroplasty. Surgical staff may be more experienced with the 3 surgical methods assessed here at high-volume centers, leading to lower perioperative costs and less time in the OR.

Currently, it is unclear which surgical method studied here has the highest clinical efficacy in treating isolated SLAP tears. A systematic review by de Sa et al.^12^ concluded that SLAP repair and biceps tenodesis are equally efficacious. However, a recently published study of active-duty military personnel found that open biceps tenodesis provides improvement in shoulder outcomes and return to activity when treating both SLAP tears and pathology of the long head of the biceps tendon.^35^ A meta-analysis performed by Ren et al.^9^ found that arthroscopic biceps tenodesis provides a better American Shoulder and Elbow Surgeons Shoulder Score (ASES) than SLAP repair. Similarly, 2 other meta-analyses found that biceps tenodesis results in greater return to sport and patient satisfaction than SLAP repair.^10^^,^^11^ Overall, it is inconclusive which method provides the best outcomes, but arthroscopic SLAP repair, arthroscopic biceps tenodesis, and open biceps tenodesis appear to all be effective treatments for SLAP tears. The increasing presence of bundled and managed care has exerted a strong pressure to lower costs. Therefore, given the increased focus on cost consciousness, it is not unreasonable for physicians to consider cost efficacy with regard to modifiable surgical factors and techniques, particularly when these techniques can be performed in a safe and skilled fashion.

### Limitations

Generalized linear models mitigate the influence of confounding variables, but limitations to the dataset still affect this study. As with any claims-based database, we are reliant on the accuracy of the coding. There may be errors in ICD-9 or CPT codes used, and some SLAP tears may have been coded with a different ICD-9 code. A larger number of arthroscopic SLAP repair cases (4,100) were compared with smaller groups of BT cases (333 open, 242 arthroscopic). Although this is in line with the prevailing preponderance of SLAP repair as opposed to BT repair, the comparative lack of BT cases reduced the power of our study. In addition, the SASD is a claims-based database and therefore may contain misclassified or miscoded data elements or cases. Furthermore, not all states code information into the SASD in the same way. For example, operative time is available only for cases performed in New York, and surgeon identifiers are available for every state except New York and Kentucky. For this reason, it is not possible to analyze the association between operative time and surgeon volume. Gaps may also exist between coding versus clinical practice. For example, some centers may not bill for every non-reusable piece of equipment expended during a case. True cost was proxied through total cost billed, but provider-insurance contracts may affect the true amount reimbursed. Additionally, costs related to revision surgery and indirect costs such as out-of-work status were not captured.

### Conclusion

In patients aged ≤50 years with isolated SLAP tears, open biceps tenodesis provides cost savings over arthroscopic methods of treatment. There was no significant cost difference between arthroscopic SLAP repairs and arthroscopic biceps tenodesis. Given the increased emphasis on cost containment, surgeons should be aware of the procedural costs associated with the treatment of SLAP tears.

## Figures and Tables

**Table A1:** Bivariate analysis of demographic and surgical variables by procedure

Procedure	Variable	Group	Percentage	Mean	95% Confidence Interval for Mean		*P* Value
					Lower Bound	Upper Bound	
Open biceps tenodesis	Facility volume	Low volume	55	$19,830	$17,745	$21,914	.045
		High volume	45	$17,009	$15,329	$18,689	
Arthroscopic SLAP repair	Postoperative admission	Not admitted	98	$21,717	$21,283	$22,151	<.001
		admitted	2	$34,777	$30,252	$39,301	
	Patient sex	Male	76	$22,267	$21,762	$22,772	.031
		Female	24	$21,130	$20,253	$22,007	
	Race	White	77	$22,155	$21,651	$22,660	<.001
		Black	8	$21,410	$19,910	$22,910	
		Hispanic	8	$26,057	$24,264	$27,849	
		Asian	1	$21,510	$17,375	$25,645	
		Other	5	$17,927	$15,936	$19,918	
	Insurance	Medicare	2	$24,968	$21,177	$28,759	.001
		Medicaid	9	$23,675	$22,104	$25,247	
		Private insurance	64	$22,179	$21,654	$22,704	
		Other	26	$20,781	$19,861	$21,701	
	Comorbidity	Not Present	64	$20,810	$20,300	$21,321	<.001
		Present	36	$24,133	$23,333	$24,934	
Arthroscopic biceps tenodesis	Patient sex	Male	66	$23,116	$20,866	$25,366	.019
		Female	34	$18,880	$16,537	$21,222	
	Facility volume	Low volume	54	$23,984	$21,347	$26,621	.003
		High volume	46	$18,969	$17,046	$20,892	

**Table A2:** Bivariate analysis of continuous variables by procedure

Procedure	Variable	Constant (SEM)	β (SEM)	*P* Value
Open biceps tenodesis	OR time	$8,045 ($2,277)	$53 ($17)	.003
Arthroscopic SLAP repair	OR time	$12,203 ($617)	$39 ($6)	<.001
	Implant	$21,299 ($267)	$1,736 ($363)	<.001
All procedures	OR time	$12,086 ($596)	$40 ($6)	<.001

OR, operating room; SEM, standard error of the mean.

**Table A3:** Bivariate analysis of demographic and surgical variables for open biceps tenodesis (BT), arthroscopic BT, and arthroscopic SLAP repair combined

Variable	Group	Percentage	Mean	Standard Deviation	95% Confidence Interval for Mean		*P* Value
					Lower Bound	Upper Bound	
Admitted	Not admitted	98	$21,512	$13,903	$21,107	$21,916	<.001
	Admitted	2	$31,140	$20,684	$27,213	$35,067	
Procedure type	Open biceps tenodesis	7	$18,598	$12,800	$17,218	$19,977	<.001
	Arthroscopic SLAP repair	88	$21,999	$14,306	$21,561	$22,437	
	Arthroscopic biceps tenodesis	5	$21,663	$13,361	$19,971	$23,355	
Patient Sex	Male	76	$22,052	$14,317	$21,580	$22,523	.008
	Female	24	$20,764	$13,705	$19,965	$21,563	
Race	White	77	$21,824	$13,942	$21,357	$22,291	<.001
	Black	8	$21,413	$13,773	$19,993	$22,832	
	Hispanic	8	$25,863	$16,482	$24,175	$27,550	
	Asian	1	$21,246	$13,361	$17,366	$25,125	
	Other	5	$18,100	$14,298	$16,274	$19,925	
Primary payer	Medicare	2	$23,856	$16,274	$20,576	$27,136	<.001
	Medicaid	9	$23,267	$15,137	$21,783	$24,751	
	Private insurance	62	$22,043	$13,615	$21,548	$22,539	
	Other	27	$20,402	$14,857	$19,585	$21,220	
≥1 comorbidity	Not present	61	$20,711	$13,301	$20,223	$21,198	<.001
	Present	39	$23,370	$15,335	$22,663	$24,077	

## Supplementary Data

ICMJE author disclosure forms
